# The association between bullying-related behaviours and subjective health complaints in late adolescence: cross-sectional study in Greece

**DOI:** 10.1186/1756-0500-7-523

**Published:** 2014-08-12

**Authors:** Spyridon Politis, Vanesa Bellou, Lazaros Belbasis, Petros Skapinakis

**Affiliations:** Department of Psychiatry, University of Ioannina School of Medicine, 451 10 Ioannina, Greece

## Abstract

**Background:**

Bullying is quite prevalent in the school setting and has been associated with several subjective health complaints such as headache, backache, abdominal pain, dizziness, fatigue and sleep problems. The aim of the present study was to investigate the association between bullying and subjective health complaints in a sample of Greek adolescents taking into account the presence of psychiatric morbidity.

**Methods:**

A stratified random sample of 2427 adolescents aged 16–18 years old and attending senior high schools were randomly selected for a computerized interview. Subjective health complaints were assessed using a symptom checklist used in the context of a previous World Health Organization study and relevant sections of the revised Clinical Interview Schedule (CIS-R). The latter was also used for the assessment of psychiatric morbidity. Bullying was assessed with the revised Olweus bully/victim questionnaire. A series of logistic regression models were used to investigate the association between bullying and subjective health complaints.

**Results:**

Victims of bullying were more likely to report backache (Odds Ratio [OR] = 1.92, 95% CI: 1.01-3.67), dizziness (OR = 2.83, 95% CI: 1.11-7.22) and fatigue (OR = 0.41, 95% CI: 0.19-0.86), independently of the presence of psychiatric morbidity. In addition bullying perpetrators were more likely to report backache (OR = 3.49, 95% CI: 1.49-8.18). It is worth noting that sleep problems and abdominal pain were also associated with being bullied and fatigue with bullying perpetration but these associations were all attenuated after adjustment for psychiatric morbidity.

**Conclusions:**

Strong associations between bullying in schools and subjective health complaints among a sample of Greek students aged 16 – 18 years have been observed. The exact nature of these associations should be investigated in future longitudinal studies.

**Electronic supplementary material:**

The online version of this article (doi:10.1186/1756-0500-7-523) contains supplementary material, which is available to authorized users.

## Background

Bullying in schools is a multifaceted problem with many consequences for all the persons involved. It occurs when a physically or socially stronger person (the perpetrator), or a group, intentionally display aggressive behaviour towards a weaker one (the victim), usually in a repetitive pattern. This kind of behaviours include verbal or physical harassment that ranges from teasing, name calling and belittling, to pushing, hitting and kicking respectively [1]. Studies that attempt to measure the prevalence of this phenomenon across the world; suggest that 20% to 30% of adolescents are involved in bullying as victims, perpetrators or both, while at school [2]. In spite of a common consensus that bullying is a worrying phenomenon that requires special attention from the scientific community, so far the actions taken towards tackling it are not sufficient [3].

Although sometimes pupils involved in bullying can overcome this experience without any harm [1], in most cases both victims and perpetrators deal with various forms of psychiatric morbidity and a number of physical effects varying from elevated levels of anxiety and depression [4–6], to physical health problems [7, 8] psychosomatic symptoms [9, 10] substance use [11] and psychiatric problems in their later adult life [12]. Even though there is a general agreement among the investigators that early intervention can prevent these harmful effects [13], studies suggest that many violent episodes in schools such as fighting and bullying goes unreported [14] and therefore the victims deprive themselves the opportunity to receive the appropriate support in order to deal with its psychological and physical health consequences [15].

Somatic symptoms have been recently examined as indicators of involvement in bullying related behaviours, mainly victimization. Somatic complains such as stomachache, headache, fatigue, sleep problems, dizziness, from school children are usually associated with factors such as school stress, anxiety and depression [16, 17], poor mental health [18] and school absenteeism [19]. It is only lately that researchers have begun to explore the relationship of such factors with violent behaviour and victimization, since in previous studies both bullying and somatic symptoms were often associated with psychiatric morbidity the first preceding [6] and the later manifesting mental health problems [18].

Such psychosomatic or functional symptoms are usually referred to as subjective health complaints (SHC), a neutral term that makes no inferences on the symptoms’ causal relationship with somatic or psychological factors. In a study investigating the association of bullying with subjective health complaints (SHC) among a sample of 11.972 Swedish adolescents, the researchers identified strong associations between SHC and bullying involvement [20]. Being a victim of bullying in school predicted an increase in somatic symptoms among sixth graders [21]. Fekkes et. al. [22] proposed that bullying often precedes the emergence of functional physical symptoms and urged health practitioners to examine the possible contribution of that experience in the appearance of such symptoms.

In the same line, emphasis has been given on the important role of the primary care physicians or pediatricians in targeting violence and victimization and on a more active engagement in identifying and resolving them [23]. Researchers consider recurrent Subjective health complaints as a clue for early detection, by school professionals, of impaired psychological and behavioural functioning [24]. More over a recent study investigated the associations between violence victims, perpetrators and somatic complaints, physical injuries and illnesses among children attending elementary schools [13]. The researchers proposed an association between involvement in bullying related behaviours with the frequency of subjective health complaints.

Regarding the situation in Greece a recent study from our team [25] reported that the prevalence of bullying related behaviours was 26.4% in a sample of 2.427 adolescents 16–18 years old attending higher secondary schools. Another study [26] using slightly different methodology reported an even higher figure of 41.3%, which brings the country in the third position over 37 countries. Smith et al. [27] attributed such elevated rates to the insufficiency of a national policy plan targeting this particular problem. Regarding subjective health complains, although there are not contemporary data concerning the prevalence of these symptoms among Greek adolescents, an earlier study [28] noted that a large number of Greek school children reported experiencing multiple subjective health complains more than once a week with the prevalence for 15 years old as high as 36% for boys and 60% for girls.

Considering the information displayed in the paragraphs above one realizes the necessity for further research into the topic and the need for implementation of national and international policies in identifying and targeting bullying related behaviours and school violence in all its forms as well as treating its short and long term consequences. The current study aimed to look for associations between bullying in schools and subjective health complaints among a particular sample of adolescent aged 16 – 18 years attending higher secondary schools in Greece. Our specific aim was to examine the association between subjective health complaints and bullying related behaviours and to examine the hypothesis that this will be independent from presence of psychiatric morbidity which is a potential confounding factor.

## Methods

### Description of the data set

We used data from the Epirus School Project [29]. This was a cross-sectional survey carried out in selected upper secondary schools in Greece with the aim to investigate the prevalence and associations of common mental disorders in late adolescence.

### Secondary education in Greece

The secondary education in Greece consists of the lower secondary schools and the upper secondary ones. The first involve the compulsory attendance of three grades (7–9) whereas the latter also involves the attendance of three grades (10–12) but with no compulsory obligation. Furthermore there is a distinction in the second upper class schools between senior high schools (Lyceum) where approximately 75% of the secondary education students attend, and technical vocational schools. In the current study the sample involved only students attending senior high schools, since this was the category surveyed in the Epirus School Project.

### Sampling of schools and pupils

The major fieldwork was undertaken for one year between January 2007 and April 2008. At the time of the design of the study approximately 75000 students attended 1193 senior high schools. The total number of schools that have been selected were 25 senior high schools including 1) all senior high schools of the major cities in the Epirus and Aetoloakarnania regions of Western/North-Western Greece, ( they have been selected due to the proximity of the selected area to the University of Ioannina), 2) the total number of senior high schools in Kalithea district (a randomly selected area of Metropolitan Athens) and 3) the total number of senior high schools of Paros Island (an Island of the Aegean Sea that has been selected due to convenience as a typical example of a school of the insular regions of Greece).

The participants included all students studying in the abovementioned schools that voluntarily agreed and consented to participate in the study (median number was 225 students per school, ranging from 138 to 425). The Ethical committee of the Ministry of Education and the Greek Educational Institute approved this study, which has been conducted in accordance with the Helsinki declaration. Consent for participation was actively obtained from both the participating students and their parents. In addition the Head of each selected School also declared its independent approval for the study.

### Design of the study and data collection procedure

A two-phase design [30] has been used in the current study. During the first phase all the participants (N = 5,614, response rate 82%) were administered in the classroom a short screening tool. This has been formulated based on the revised Clinical Interview Schedule (CIS-R) a complete structured diagnostic instrument used in the study’s second phase. The participants were also asked a number of sociodemographic questions.

The selection of the participants for the second phase involved a stratified random sampling procedure according to the scores obtained in the administration of the short screening instrument. In particular, all of the participants with high scores (>75^th^ percentile), 30% of the participants with middle scores and 10% of the ones that scored low (<25^th^ percentile), have been included. An exemption was made in the two schools of Paros Island where all consenting pupils completed the computerized interview due to the availability of local fieldworkers (therefore the two phases were merged into one in Paros island).

The second phase was carried out in the schools’ IT laboratories. There the participants (N = 2431, response rate 95%) completed the computerized interview consisting of the full CIS-R interview and other relevant questions for bullying and subjective health complaints. In the final analysis 2427 students were used since from the initial number of the participants (N = 2431) four of them had missing values regarding the sociodemographic question items, administered in the first phase of the study.

### Assessment of psychiatric morbidity: the revised clinical interview schedule (CIS-R)

Psychiatric symptoms were assessed with the revised clinical interview schedule (CIS-R), a fully structured psychiatric interview designed to be used by trained lay interviewers [31]. The CIS-R was the main instrument used in the national psychiatric morbidity surveys in the UK [32] and has been used in several other similar surveys around the world. A computerized version has also been developed and found to be comparable with the regular interview [33]. The CIS-R was originally designed to assess symptoms in participants above 16 years old but has been previously used in teenagers above 14 years old in Australia [34]. The CIS-R assesses the presence and severity of common psychological symptoms (somatic symptoms, fatigue, concentration/memory problems, sleep problems, irritability, depressive mood, depressive ideas, general worry, worry about physical health, free-floating anxiety, phobias, panic anxiety, compulsions and obsessions). Two screening questions in each section ask about the presence of the symptom during the past month and then there is a more detailed assessment of the presence, frequency, duration and severity of the symptom during the past seven days. Each symptom section is scored on a scale ranging from 0 to 4 (except depressive ideas scored from 0 to 5).

The Greek version of the CIS-R was translated and back-translated using the procedure recommended by the World Health Organization (http://www.who.int/substance_abuse/research_tools/translation/en/index.html). The psychometric properties of the Greek version of the CIS-R including its factor structure and internal consistency have been reported elsewhere [29]. An internal consistency reliability analysis showed that item-test correlations ranged from 0.42 to 0.74, item-rest correlations ranged from 0.30 to 0.67 and Cronbach’s alpha ranged from 0.84 to 0.87 with an overall alpha for CIS-R of 0.86. A test-retest reliability of the CIS-R was carried out in a subset of the present data set (two schools of the city of Ioannina with an interval between assessments of 2 weeks) and was found to be 0.84. [29]. Possible scores ranging from 0–57, with scores of 12 or above on the CIS-R indicating clinically psychiatric morbidity [29, 35] a score of 6–11 indicates sub-threshold symptoms of mental disorder, a score of 0–5 indicates little evidence of mental disorder [35], while a score of 18 or more has been used as an indicator of severe psychiatric morbidity [36]. For the purposes of the present paper, we have used the total CIS-R score as an indicator of psychiatric morbidity. Since two of the sections of the CIS-R were used to assess the somatic symptoms of fatigue and sleep problems (see below the relevant section) we have excluded from the total score the scores on these two sections (therefore the range of scores is from 0 to 49 for the remaining 12 sections).

### Assessment of school bulling

Involvement in bullying either as a perpetrator (bully others) or as a victim (being bullied by others) was investigated in the second phase of the study. We used two questions, one for being bullied and one for bullying others, taken from the revised Olweus Bully/Victim Questionnaire [37] which was also used in a WHO youth health study [38]. An introductory sentence defined bullying as follows:

*“The next questions are about bullying. We say a pupil is being bullied when another pupil, or a group of pupils, says or does nasty and unpleasant things to him or her. It is also bullying when a pupil is teased repeatedly in a way he or she doesn’t like. But it is not bullying when two pupils of about the same strength quarrel or fight.”*

The respondents were further asked how frequently they had been bullied or had bullied others during the last 2 months in school. The possible answers were: “many times a week”, “about once a week”, “2 or 3 times per month”, “1 or 2 times during the last 2 months” and “not at all”. If the participant had been involved in this behaviour at least once a week, this was classified as “frequent” bullying or victimization respectively, whereas all other instances were classified as “less frequent” bullying or victimization. We based our main analyses on the “frequent” group only, as the less frequent type is not universally accepted as pathological.

However, in order to provide a more detailed analysis of our data, we performed supplementary analyses including also the less frequent category “2 or 3 times per month” as suggested by Solberg et al. [39], who consider this category a reasonable cut-off point (see our appendix in Additional file 1).

### Assessment of somatic symptoms

We assessed 6 somatic symptoms backache, headache, abdominal pain, dizziness fatigue and sleep problems. The presence and frequency of the somatic symptoms was investigated in the second phase of the study. For the first 4 symptoms we selected the corresponding items from the symptom checklist used in the context of the WHO study “Health behaviour in school-aged children (HBSC)” [40]. Students were asked to report how often they had experienced any of the following symptoms: abdominal pain, backache, headache, dizziness. There were five possible answers, “about every day”, “at least once a week”, “at least once every 2 weeks”, “at least once a month” and “rarely or never”. We classified these symptoms as clinically significant when present every day.

The presence of fatigue and sleep problems were assessed by selecting the corresponding items in the relevant sections of the CIS-R. In particular students were asked to report if they experienced fatigue and/or sleeping problems during the past month. Then a more detailed assessment of the presence, frequency, duration and severity of the symptom during the past seven days took place. With scores ranging in every section from 0–4, we classified these symptoms as clinically significant when the participants scored 2 or above [31].

### Other variables

Information about several socioeconomic and sociodemographic variables was obtained from the students in the first phase of the study (own age, parent’s age, gender, parent’s marital status, number of brothers and sisters, mother’s educational status, father’s educational status, mother’s employment status, and father’s employment status).

### Statistical analyses

The analyses were all conducted using the statistical software package STATA 10.0 (StataCorp, College Station, Texas). The associations between bullying and psychiatric morbidity, socioeconomic and other variables were investigated using logistic regression models. To take into account the potential effect of clustering of our data (since adolescents were nested into 25 schools) we first carried out a two-level logistic model (level 1: individuals, level 2: schools) in Stata using the gllamm command [35]. We also performed the models with the survey commands of Stata (svylogit) using school as the stratum. Results were very similar with both models and therefore in the paper we present the results using the survey commands because their use is more widespread in the literature. It should be noted that the effect of schools was negligible with an intraclass correlation coefficient close to zero. In all analyses we have used probability weights to take into account the stratified random sampling procedure.

## Results

### Description of the sample

Overall 5614 students took part in the first phase of the study (55% girls, 41% 10^th^ grade, 31% 11^th^ grade, 28% 12^th^ grade), while 2431 students were interviewed in the second phase (59% girls, 39% 10^th^ grade, 32%11^th^ grade, 29% 12^th^ grade). A detailed table of the sociodemographic characteristics of the whole sample in both phases of the study is given in the appendix (additional file 1: Table A1). Due to the stratified sampling procedure there were more female than male students in the second phase.

### Prevalence of bullying behaviours and subjective health complaints

Table 1 presents the prevalence of bullying-related behaviours by gender. Regarding frequent bullying, 1.4% of the students (boys: 1.5%, girls: 1.3%) reported frequent victimization and 2.8% (boys: 4.8%, girls: 0.7%) frequent perpetration. It can be seen that boys were more likely to report that they had bullied others compared to girls. In contrast, there was no gender difference in victimization.

**Table 1:** **Prevalence of bullying-related behaviours in Greek adolescents 16–18 years old attending senior high schools (N = 2427)**

	Male	Female	Total
	N (%) ^1^	N (%) ^1^	N (%) ^1^
**«Bullied by others» - Victims**			
Not at all	836 (87.2%)	1238 (89.3%)	2074 (88.2%)
Less frequent victimisation (<weekly)	130 (11.3%)	173 (9.4%)	303 (10.4%)
Frequent victimization (at least weekly)	22 (1.5%)	28 (1.3%)	50 (1.4%)
	p = 0.41		
**«Bullying others» - Perpetrators**			
Not at all	694 (72.4%)	1274 (89.1%)	1968 (80.7%)
Less frequent bullying others (<weekly)	240 (22.8%)	151(10.1%)	391 (16.5%)
Frequent bullying others (at least weekly)	54 (4.8%)	14 (0.7%)	68 (2.8%)
	p < 0.001		

^1^Actual number of observations; percentages in comparison are weighted to take into account the stratified random sampling procedure. Chi-square test was performed to examine sex differences.

Table 2 presents the prevalence of Greek adolescents (16–18 years old) reporting clinically significant subjective health complaints, by gender. Among them 9% (boys: 6%, girls: 11%) reported backache, 10% (boys: 4%, girls: 16%) reported headache, 6% (boys: 3%, girls 9%) reported abdominal pain, 3% (boys: 2%, girls: 5%) reported dizziness, 37% (boys: 31%, girls: 43%) reported fatigue and 16% (boys: 14%, girls: 19%) sleep problems. It can be seen that girls were more likely to report subjective health complaints compared to boys.

**Table 2:** **Prevalence of Greek adolescents (16–18 years old) reporting clinically significant subjective health complaints (N = 2427)**

	Male	Female	Total
	N (%) ^1^	N (%) ^1^	N (%) ^1^
**Backache**	88 (6%)	195(11%)	283 (9%)
P < 0.001			
**Headache**	71 (4%)	289 (16%)	360 (10%)
P < 0.001			
**Abdominal pain**	44 (3%)	187 (9%)	231 (6%)
P < 0.001			
**Dizziness**	32 (2%)	124 (5%)	156 (3%)
P < 0.001			
**Fatigue**	399 (31%)	814 (43%)	1213 (37%)
P < 0.001			
**Sleeping problems**	169 (14%)	370 (19%)	539 (16%)
P = 0.004			

^1^Actual number of observations; percentages in comparison are weighted to take into account the stratified random sampling procedure. Chi-square test was performed to examine sex differences.

### Associations between bullying victims and subjective health complaints

Odds ratios and 95% confidence intervals for the associations of victimization due to bullying with complaints for somatic symptoms before and after adjustment for sociodemographic/socioeconomic factors and psychiatric morbidity are shown in Table 3.

**Table 3:** **Adjusted odds ratios of being a victim for several subjective health complaints and psychiatric morbidity in adolescents 16–18 years old attending senior high schools in Greece (N = 2427)**

	Victims (Bullied by others at least weekly)		
	OR (95% CI)		
	Model 1	Model 2	Model 3
	Adjusted for gender and age	Adjusted for all sociodemographic factors ^1^	Adjusted for sociodemographic factors and psychiatric morbidity
**Subjective health complaints**			
Backache	**3.94 (1.32–1.72)**	**3.62 (1.63-8.03)**	**1.92 (1.01-3.67)**
Headache	1.89 (0.91-3.91)	1.87 (0.86-4.10)	0.68 (0.27-1.71)
Abdominal pain	**4.66 (1.15-18.95)**	**3.91 (1.62-9.40)**	1.42 (0.62-3.24)
Dizziness	**8.59 (2.23-33.13)**	**7.01 (2.90-16.92)**	**2.83 (1.11 -7.22)**
Fatigue	1.42 (0.71-2.84)	1.35 (0.70-2.62)	**0.41 (0.19-0.86)**
Sleep problems	**3.12 (1.30-7.52)**	**3.02 (1.43-6.38)**	1..69 (0.75-3.80)
**Psychiatric symptoms as assessed by the CIS-R**	**-**	**-**	**1.06 (1.01 -1.10)**

^1^Sociodemographic factors included own age, parent’s age, gender, parent’s marital status, number of brothers and sisters, mother’s educational status, father’s educational status, mother’s employment status, and father’s employment status.

*OR* Odds ratio, *CI* Confidence Interval, *CIS-R* score on the revised Interview Schedule.

Figures in bold statistically significant at p<0.05.

The statistical analysis revealed positive associations between bullying victimization and the experience of subjective health complaints among the victims.

In particular being bullied by others was associated with experiencing somatic symptoms such as backache (OR = 1.92, 95% CI: 1.01-3.67), dizziness (OR = 2.83, 95% CI: 1.11-7.22) and fatigue (OR = 0.41, 95% CI: 0.19-0.86). Victims were also more likely to report abdominal pain and sleep problems, although this association diminished after adjustments for psychiatric morbidity. Moreover victims were more likely to experience psychiatric symptoms (OR = 1.06, 95% CI: 1.01-1.10).

Considering the additional analyses we performed including also the category 2 or 3 times per month (less frequent /frequent) the results showed that bullying victims were more likely to experience headache (OR = 0.62, 95% CI: 0.40-0.97) and dizziness (OR = 2.16, 95% CI:1.28-3.65) as well as experiencing psychiatric symptoms (OR = 1.05, 95% CI: 1.04-1.07) ( see Additional file 1: Table A2).

### Associations between bullying perpetrators and subjective health complaints

Odds ratios and 95% confidence intervals for the associations of bullying perpetrators with complaints for somatic symptoms before and after adjustment for sociodemographic/socioeconomic factors and psychiatric morbidity are shown in Table 4.

**Table 4:** **Adjusted odds ratios of being a perpetrator for several subjective health complaints and psychiatric morbidity in adolescents 16–18 years old attending senior high schools in Greece (N = 2427)**

	Perpetrators (Bullying Others at least weekly)		
	OR (95% CI)		
	Model 1	Model 2	Model 3
	Adjusted for gender and age	Adjusted for all sociodemographic factors ^1^	Adjusted for all sociodemographic factors and psychiatric morbidity
**Subjective health complaints**			
Backache	**3.70 (1.60 – 8.55)**	**4.15 (1.74-9.93)**	**3.49 (1.49-8.18)**
Headache	1.15 (0.56-2.39)	1.25 (0.50-3.13)	0.64 (0.23-1.77)
Abdominal pain	1.73 (0.64-4.66)	**3.19 (1.02-9.95)**	2.04 (0.57-7.30)
Dizziness	1.12 (0.39-3.23)	1.40 (0.48-4.10)	0.42 (0.11-1.60)
Fatigue	**3.05 (1.55-6.04)**	**2.94 (1.57-5.51)**	2.02 (0.97-4.22)
Sleep problems	1.89 (0.89-4.00)	1.71 (0.84-3.51)	1.05 (0.50 – 2.22)
**Psychiatric symptoms as assessed by the CIS-R**	**-**	**-**	**1.04 (1.02 -1.07)**

^1^Sociodemographic factors included own age, parent’s age, gender, parent’s marital status, number of brothers and sisters, mother’s educational status, father’s educational status, mother’s employment status, and father’s employment status.

*OR* Odds ratio, *CI* Confidence Interval, *CIS-R* score on the revised Interview Schedule.

Figures in bold statistically significant at p<0.05.

Bullying perpetrators were more likely to report backache (OR = 3.49, 95% CI: 1.49-8.18). They were also more likely to report fatigue although this association marginally diminished (OR = 2.02, 95% CI: 0.97- 4.22, p = 0.06) after adjustments for psychiatric morbidity. Psychiatric morbidity also showed a significant association with being a perpetrator.

Considering the additional analyses we performed including also the category 2 or 3 times per month (less frequent /frequent) the results did not reveal any associations between bullying others and subjective health complaints. Being a perpetrator were only associated with psychiatric morbidity (OR: 1.03, 95% CI: 1.01-1.04) (see additional file 1: Table A3)

## Discussion and conclusions

### Main findings

The current cross-sectional study looked for associations between bullying in schools and subjective health complaints among a sample of Greek adolescent students after adjustments for socioeconomic, sociodemographic factors and psychiatric morbidity. There was evidence that being a bullying victim was independently associated with experiencing a number of subjective health complaints such as backache, dizziness and fatigue. In addition bullying perpetrators were more likely to report suffering from backache. We should also mention that sleep problems and abdominal pain were also associated with being bullied and fatigue with bullying perpetration but these associations were all attenuated after adjustment for psychiatric morbidity.

### Comparison with other studies

As stated above our results suggest a noteworthy association between bullying and health issues such as functional somatic disturbances among Greek adolescents, and add to the relatively limited research evidence [13] concerning these associations. Although the prevalence of bullying varies considerably among different countries [36], our results, regarding bullying prevalence, are in line with several studies that have been put forward and used similar methodology [41]. What is more, an association was reported between experiencing subjective health complains and both bullying perpetration and victimization, after adjustments for sociodemographic, socioeconomic factors and psychiatric morbidity. Similar associations have been described by previous studies [8, 13, 20, 22, 23]. Most of the previous studies have mainly concentrated in victims of bullying behaviour and have not looked at the perpetrators group as we did in the present study.

In particular, according to our results bullying victimization was associated with somatic symptoms such as dizziness, backache and fatigue. Additionally, bullying victims were more likely to report abdominal pain and sleep problems although this association weakened when adjusted for psychiatric morbidity. Our findings come to support other researches’ [8, 13, 20, 22, 23] evidence suggesting that, somatic symptoms are associated with victimization. Nevertheless, due to the cross-sectional nature of our study we cannot draw causal relationships between our variables.

However in this study it became apparent that regardless of causality somatic symptoms are often indicators of victimization. These finding add to our understanding of violence and victimization’s association and contributes to our knowledge about how to point out victimization and prevent its harmful effects by intervening as early as possible [13]. At the same time our findings raise the necessity for further research concerning the causal relations between somatic symptoms and victimization.

In addition among a number of subjective health complaints, backache stood out as a significant health complaint associated with bullying perpetration. Fatigue was also associated with a greater likelihood of bullying others but this association was marginally attenuated after adjustment for psychiatric morbidity. Our findings confirm previous studies (8, 13, 22) that pinpoint the association between somatic symptoms and bullying perpetration. Perpetrators also deal with physical problems and this raises the need to investigate the paths through which these problems come about. Researchers [13] suggest that stressful, prolonged violent interactions can weaken the immune system of the child involved through a physiological route. In any case further research is necessary. In our study although the interpretation of this association is difficult given the cross-sectional nature, it is nevertheless important to highlight it as it can make apparent possible involvement in bullying behaviour.

### Limitations of the study

When interpreting the above mentioned findings the cross-sectional nature of our study should be taken into account, since it does not allow us to make any causal inference about the association between bullying and the Subjective Health Complaints studied.

Participation in bullying either as victim or a perpetrator as well as their health status was assessed through participant’s self reports. Relying on self report data bears the risk of information bias. An alternative could be to base our measures from different sources such as peer reports. Nevertheless research findings in this topic from studies using self reports, display a high consistency [42–45]. Our sample of schools was not random and therefore selection bias cannot be ruled out although it is unlikely. In addition, our sample did not include adolescents attending technical vocational schools but only those attending senior high schools. This limits the generalizability of our results.

### Implications

This study explored the associations of bullying with subjective health complaints. Our study shows that there are associations between bullying related behaviours and specific subjective health complaints. This finding is important and facilitates the identification of problematic behaviours considering the reluctance on the parts of the students to disclose their potentially traumatic experiences [15]. Early intervention strategies for the health needs of the victims and the perpetrators may be important [13, 20]. In addition the outcome of the current research shows that bullying is associated with unpleasant health defeating experiences not just for the victims but for all the parties involved and this raises the necessity for further research.

## Electronic supplementary material

Additional file 1: **Politis et al. Bullying-related behaviours and subjective health complaints.** (DOCX 18 KB)
