# Supplementary material for: The association between bullying-related behaviours and subjective health complaints in late adolescence: cross-sectional study in Greece
Source: BMC Res Notes. 2014 Aug 12;7:523. doi: 10.1186/1756-0500-7-523 (PMC4267138; doi:10.1186/1756-0500-7-523)
Supplement: Supplementary file 1 — Additional file 1: Politis et al. Bullying-related behaviours and subjective health complaints. (DOCX 18 KB) [file 13104_2013_3044_MOESM1_ESM.docx]

## Politis et al. Bullying-related behaviours and subjective health complaints

## Additional File 1

**Table A1**. Basic Description of the Sample in the two phases of the study

| **Variable** | **Phase 1 (n=5614)** | **Phase 2 (N=2431)** |
| --- | --- | --- |
| **Gender**  Male  Female | 2530 (45%)  3084 (55%) | 989 (41%)  1442 (59%) |
| **Age**  16  17  18 | 2265 (41%)  1869 (33%)  1440 (26%) | 957 (40%)  825 (34%)  627 (26%) |
| **Grade**  10^th^ grade  11^th^ grade  12^th^ grade | 2281 (41%)  1772 (31%)  1561 (28%) | 942 (39%)  778 (32%)  711 (29%) |
| **Parent’s Family Status**  Married  Divorced / Separated  Widow  Missing | 5012 (89%)  394 (7%)  159 (3%)  49 (1%) | 2145 (88%)  178 (7.5%)  82 (3.5%)  26 (1%) |
| **Father’s Employment**  Employed – Public Sector  Employed – Private Sector  Self-employed  Unemployed  Retired  Other/Missing | 1828 (33%)  1183 (22%)  1949 (35.5%)  42 (0.5%)  290 (5%)  224 (4%) | 796 (33%)  511 (21%)  839 (34.5%)  17 (0.5%)  123 (5%)  145 (6%) |
| **Mother’s Employment**  Employed – Public Sector  Employed – Private Sector  Self-employed  Looks after House  Unemployed  Other/Missing | 1679 (30%)  1056 (19%)  741 (13%)  1720 (31%)  234 (4%)  184 (3%) | 681 (28%)  446 (18.5%)  347 (14%)  748 (31%)  109 (4.5%)  100 (4%) |
| **Father’s Educational Status**  Primary  Secondary Basic  Secondary Complete  Technological degree  University degree | 791 (14%)  849 (15%)  1589 (29%)  738 (13%)  1562 (28%) | 382 (16%)  344 (14%)  733 (30%)  309 (13%)  663 (27%) |
| **Mother’s Educational Status**  Primary  Secondary Basic  Secondary Complete  Technological degree  University degree | 743 (13%)  784 (14%)  2086 (37.5%)  584 (10.5%)  1385 (25%) | 338 (14%)  365 (15%)  915 (37.5%)  233 (9.5%)  580 (24%) |

## Table A2. Adjusted odds ratios of being a victim for several subjective health complaints and psychiatric morbidity in adolescents 16-18 years old attending senior high schools in Greece (N=2427).

|  | **Victims (Bullied by Others at least 2 or 3 times per month)**  OR (95% CI) | | |
| --- | --- | --- | --- |
|  | **Model 1**  Adjusted for gender and age | **Model 2**  Adjusted for all sociodemographic factors^1^ | **Model 3**  Adjusted for all sociodemographic factors and psychiatric morbidity |
| **Subjective Health Complaints**  Backache  Headache  Abdominal pain  Dizziness  Fatigue  Sleep Problems | **1.66 (1.01-2.74)**  1.31 (0.91 – 1.88)  **2.34 (1.40-3.91)**  **4.08 (2.39 – 6.97)**  **1.78 (1.31 – 2.43)**  **1.60 (1.11-2.31)** | 1.11 (0.69-.79)  0.75 (0.49-1.16)  1.51 (0.96-2.39)  **2.74 (1.67-4.49)**  **1.15 (1.03-1.29)**  1.14 (0.98-1.33) | 1.12 (0.68-1.82)  **0.62 (0.40-0.97)**  1.28 ( 0.79-2.07)  **2.16 (1.28-3.65)**  0.98(0.86-1.11)  1.03 (0.87-1.23) |
| **Psychiatric Symptoms as assessed by the CIS-R** | **-** | **-** | **1.05 (1.04-1.07)** |

^1^Sociodemographic factors included own age, parent's age, gender, parent's marital status, number of brothers and sisters, mother's educational status, father's educational status, mother's employment status, and father's employment status.

OR: Odds ratio; CI: Confidence Interval; CIS-R: score on the revised Interview Schedule

## Table A3. Adjusted odds ratios of being a perpetrator for several subjective health complaints and psychiatric morbidity in adolescents 16-18 years old attending senior high schools in Greece (N=2427).

|  | **Perpetrators (Bullying Others at least 2 or 3 times per month )**  OR (95% CI) | | |
| --- | --- | --- | --- |
|  | **Model 1**  Adjusted for gender and age | **Model 2**  Adjusted for all SES and sociodemographic factors^1^ | **Model 3**  Adjusted for all SES, sociodemographic factors and psychiatric morbidity |
| **Subjective Health Complaints**  Backache  Headache  Abdominal Pain  Dizziness  Fatigue  Sleep Problems | 1.27 (0.79-2.05)  1.49 (0.98-2.25)  1.37 (0.85-2.21)  1.05 (0.58-1.91)  **1.51 (1.13-2.03)**  1.22 (0.87 -1.70) | 1.04 (0.61-1.78)  1.37 (0.87-2.15)  1.18 (0.72-1.95)  0.74 (0.37-1.49  1.10 (0.98-1.23)  1.00 (0.87-1.14) | 1.04 (0.61-1.76)  1.24 (0.78-1.97)  1.07 (0.65-1.77)  0.64 (0.32-1.28)  1.02 (0.89-1.16)  0.94 (0.82-1.09) |
| **Psychiatric Symptoms as assessed by the CIS-R** | _ | - | **1.03 (1.01-1.04)** |

^1^Sociodemographic factors included own age, parent's age, gender, parent's marital status, number of brothers and sisters, mother's educational status, father's educational status, mother's employment status, and father's employment status.

OR: Odds ratio; CI: Confidence Interval; CIS-R: score on the revised Interview Schedule
